# Genome-Wide Identification of Transcription Start Sites in Two *Alphaproteobacteria*, Rhodobacter sphaeroides 2.4.1 and Novosphingobium aromaticivorans DSM 12444

**DOI:** 10.1128/MRA.00880-20

**Published:** 2020-09-03

**Authors:** Kevin S. Myers, Jessica M. Vera, Kimberly C. Lemmer, Alexandra M. Linz, Robert Landick, Daniel R. Noguera, Timothy J. Donohue

**Affiliations:** a Great Lakes Bioenergy Research Center, Wisconsin Energy Institute, University of Wisconsin—Madison, Madison, Wisconsin, USA; b Center for High Throughput Computing, University of Wisconsin—Madison, Madison, Wisconsin, USA; c Department of Biochemistry, University of Wisconsin—Madison, Madison, Wisconsin, USA; d Department of Bacteriology, University of Wisconsin—Madison, Madison, Wisconsin, USA; e Department of Civil & Environmental Engineering, University of Wisconsin—Madison, Madison, Wisconsin, USA; University of Southern California

## Abstract

Here, we report the genome-wide identification of transcription start sites (TSSs) from two *Alphaproteobacteria* grown under conditions that result in significant changes in gene expression. TSSs that were identified as present in one condition or both will be an important resource for future studies of these, and possibly other, *Alphaproteobacteria*.

## ANNOUNCEMENT

Rhodobacter sphaeroides and Novosphingobium aromaticivorans are metabolically diverse and industrially relevant *Alphaproteobacteria*. R. sphaeroides is a facultative bacterium that can harvest solar energy, fix nitrogen, sequester CO_2_, and produce valuable chemicals (1–5), while *N. aromaticivorans* can convert aromatics found in contaminated environments, or derived from lignin, into bioproducts (6–9). Recently, genome-scale experiments have been performed to better understand the metabolic and regulatory networks of each organism, including an analysis of protein-DNA interactions (2, 10–13), global transcript abundance measurements (8, 10, 11, 13–16), and identification of conditionally essential genes using transposon-based sequencing of mutant libraries (9, 17). Here, we report on genome-wide transcription start site (TSS) identification using high-throughput sequencing (TSS-seq) during aerobic respiration and anaerobic photosynthetic growth of R. sphaeroides in Sistrom’s medium (18) at 30°C during mid-log phase and during aerobic growth of *N. aromaticivorans* in the presence and absence of the aromatic compound vanillic acid in modified Sistrom’s medium (8, 18) at 30°C during mid-log phase.

Three replicates of R. sphaeroides 2.4.1 or *N. aromaticivorans* DSM 12444 *ΔsacB* cultures were grown, and RNA was isolated as previously described (8, 18, 19). TSS-seq libraries were produced using RppH, which converts the 5′ triphosphates on unprocessed mRNA species to monophosphates, making them a substrate for ligation of the Illumina adapters (20). The resulting material was sequenced on an Illumina HiSeq 2500 instrument (1 × 50 bp; 117,189,686 total reads for R. sphaeroides and 63,260,190 total reads for *N. aromaticivorans*) (Table 1). The FASTQ files were split using the index barcode sequences to separate the sequences for the samples treated with or without RppH (RppH^+^ and RppH^−^, respectively) using fastx_barcode_splitter.pl version 0.0.13.2 (http://hannonlab.cshl.edu/fastx_toolkit/). The sequences were trimmed to remove any remaining adapter-derived bases using Trimmomatic version 0.3 (HEADCROP, 6; MINLEN, 25) (19) and were aligned to the R. sphaeroides genome (assembly ASM1290v2, GenBank accession number GCF_000012905.2) or the *N. aromaticivorans* genome (assembly ASM1332v1, GenBank accession number GCF_000013325.1) using Bowtie 2 version 2.3.5.1 (21), allowing for one mismatch (38,571,087 total aligned reads for R. sphaeroides and 29,552,504 total aligned reads for *N. aromaticivorans*) (Table 1). The aligned Bowtie 2 file was further processed with Picard tools version 2.10.0 (https://broadinstitute.github.io/picard/) and SAMtools (22). The genomeCov command from BEDtools version 2.27.0 (https://bedtools.readthedocs.io/en/latest/) was used to identify genomic locations of the first base in each aligned sequence read, which we defined as the TSS. A pseudocount of 1 was added to all TSS read values to prevent division by 0. The R package edgeR (version 3.10) (23) was used to map locations with a statistically significant increase in read abundance in the RppH^+^ samples compared to the RppH^−^ samples. Locations with a significant increase in read count in the RppH^+^ samples compared to the RppH^−^ samples (false discovery rate [FDR], ≤0.05) were retained, defined as TSSs, and associated with genes if the TSS was 350 bp upstream of the translation start site.

**TABLE 1:** Summary of sequencing statistics for each sample

Sample^*a*^ by bacterial species	Total no. of sequence reads	No. of trimmed sequence reads	No. of aligned sequence reads
R. sphaeroides			
Aerobic Rep A RppH^−^	8,254,464	6,065,679	2,803,014
Aerobic Rep A RppH^+^	8,383,862	6,080,322	2,795,258
Aerobic Rep B RppH^−^	6,174,801	4,622,659	2,456,095
Aerobic Rep B RppH^+^	8,457,109	6,270,320	3,469,770
Aerobic Rep C RppH^−^	11,058,996	8,247,098	3,089,246
Aerobic Rep C RppH^+^	13,653,189	10,112,149	4,366,100
Photosynthetic Rep A RppH^−^	10,038,077	7,340,387	1,565,952
Photosynthetic Rep A RppH^+^	11,451,252	8,654,668	4,121,559
Photosynthetic Rep B RppH^−^	7,016,230	4,765,608	1,914,259
Photosynthetic Rep B RppH^+^	8,489,377	6,181,210	3,726,327
Photosynthetic Rep C RppH^−^	11,377,067	8,247,308	2,631,811
Photosynthetic Rep C RppH^+^	12,835,262	9,648,342	5,631,696
*N. aromaticivorans*			
Glucose Rep A RppH^−^	6,147,801	4,690,677	2,542,350
Glucose Rep A RppH^+^	5,912,261	4,531,213	2,262,352
Glucose Rep B RppH^−^	4,557,911	3,387,765	2,286,253
Glucose Rep B RppH^+^	4,706,281	3,498,597	2,236,403
Glucose Rep C RppH^−^	5,300,718	4,054,452	2,728,261
Glucose Rep C RppH^+^	5,099,093	3,891,366	2,492,722
Vanillic Acid Rep A RppH^−^	3,673,324	2,808,993	1,596,876
Vanillic Acid Rep A RppH^+^	4,952,555	3,789,825	2,709,442
Vanillic Acid Rep B RppH^−^	3,773,367	2,873,551	1,725,337
Vanillic Acid Rep B RppH^+^	5,212,702	3,992,016	3,044,911
Vanillic Acid Rep C RppH^−^	6,280,041	4,754,420	2,122,011
Vanillic Acid Rep C RppH^+^	7,644,055	5,887,229	3,805,586

a Each sample was split and treated either with (RppH^+^) or without (RppH^−^) RppH as described (20).

**FIG 1:**
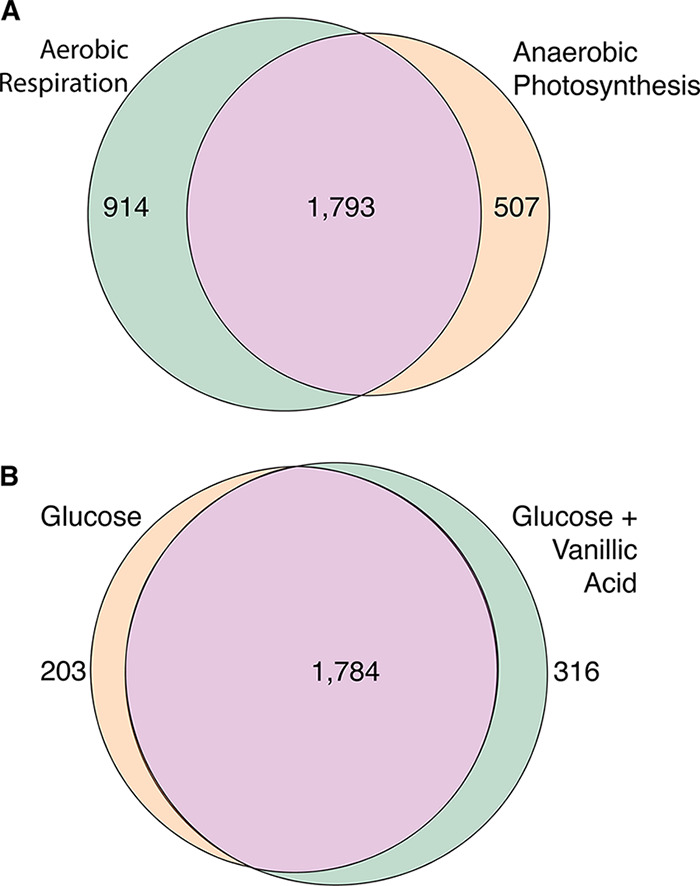
Condition-dependent transcription start site (TSS) identification. TSS populations from R. sphaeroides grown by aerobic respiration and anaerobic photosynthetic conditions (A) and *N. aromaticivorans* grown in glucose and glucose plus vanillic acid (B). The differences in TSSs in R. sphaeroides and *N. aromaticivorans* provide a new molecular view on previous reports of condition-dependent changes in gene expression in these *Alphaproteobacteria* (9, 24–26).

In total, 3,214 unique TSSs were identified from the two R. sphaeroides conditions, with 1,793 common TSSs, supporting a large core of promoters used under both conditions and a dramatic reprogramming of the transcriptional network under the two conditions (Fig. 1) (24–26). Of the 2,303 unique TSSs identified under the two *N. aromaticivorans* conditions, 1,784 were common to both growth conditions, suggesting that there is also a significant transcriptional reprogramming in the presence of an aromatic substrate (Fig. 1). These TSS data sets will serve as a valuable resource to the community, aiding in defining transcription units, identifying promoter elements, predicting binding sites for sigma and other transcription factors, and helping test predictions on the genome-scale metabolic and transcriptional changes associated with lifestyle changes in these and possibly other bacteria (9).

### Data availability.

Data are publicly available at NCBI GEO (GSE150944) and SRA (SRP245572).
